# Blount on Opium

**Published:** 1860-04

**Authors:** J. Blount

**Affiliations:** Rockford, Ill.


### ARTICLE 4.

ROCKFORD, Ill., March 1860.

*Editors of the Chicago Medical Journal,*

DEAR SIRS :

In the January number of your Journal I was happy to find Prof. Hudson's "Prize Essay on opium," in its "connected and more intelligible form."

If Prof. Hudson "enters the averment of a *stout denial* to the teachings of the great English author, Pereira," I trust he will pardon an humble member of the profession for offering a *small criticism* to one part of his Essay.

It is this, found on page 41 of the Journal: "a *convenient* and *safe* rule for the administration of opium to infants under a year old, is to give a drop of officianal laudanum for every *month* in the child's age." (The italicizing is mine.)

When I read the above quotation, I supposed it was an "*imperfect print*," but finding no correction in the February number of the Journal, I can understand it *only* as "the text of the undisturbed deliberations of the Essayist," and could not let it pass without notice.

When I was a student I was told Pereira was good authority. Acting upon that teaching I purchased his work, and have ever since been pleased with it. Turning to article Opium, under the head of Tr. Opii, or Laudanum, I find, "for an adult (the dose of laudanum) varies from minims x to f ʒ i. To *children* it must be given with the *greatest* caution. I have seen a powerful effect produced in a very young infant by *one* drop." Other authors speak of the *medium* dose of laudanum as *twenty* drops for an adult. *All*, or at least *most* writers agree that infants do *not* bear opium in the same proportional dose, that they do most other remedies, compared with adults. A person 20 years old, may, for practical purposes, be called an adult. Now if an infant twelve months (1 year) old, will "*safely* and *conveniently*" bear twelve drops of laudanum, then a person 20 years old will bear, at least, twenty times as much, two-hundred and forty drops, or f ʒ iv, or twelve grains of solid opium. Well, I know adults *will* bear such a dose of opium, for I have administered it. But, that is not granting it is a "*safe* and *convenient*" dose, meaning, of course, an *ordinary* dose. It would be only in *extreme* cases I would venture upon such *heroic* treatment. On page 39 of Journal I find, "If an infant is tender and susceptible, it is therefore more susceptible to medicines, a responsiveness which holds to the advantage of the discreet physicians. It then requires a less dose," but infants do *not* bear the same proportional dose of laudanum with adults, so I think twelve drops of laudanum to an infant one year old would be equal to f ʒ vi, or equal eighteen grains of solid opium to an adult. And on page 41 I find, "The poisonous dose does not appear to be reached *much short* of *twenty grains*. A *few* may be poisoned with a *less* dose, but many will escape the toxic influence of a much larger quantity." Now, if (as I think has been fairly shown) that Essay teaches eighteen grs. of opium a "*safe* and *convenient*" dose for an adult, I think it comes mighty near to a poisonous dose, so near, it should be administered *only* by the most *careful* hand, guarded by a most accurately adjusted balance. It *is* too near a toxic dose to be "*safe* and *convenient*."

Pereira says of the dose of opium, "one-eighth to one-half a grain is a *small dose* for an adult, from half a gr. to two grs. we term a *medium* dose, and from two to five grs. we denominate a *full* or *large dose*."

While *I* would without hesitancy far exceed the dose as given by Pereira as a "*full* or *large* dose" in extreme cases, (and I doubt not *he* would too) I can but depricate the open handedness with which it is dealt in the Essay.

On page 42 a case is reported, as being rather remarkable in which "a child not quite six years old took *seven* and a *half* grs. of opium without a fatal effect." Now if we figure on that case, we find that a child one year old will take *twelve* drops of laudanum as a "*safe* and *convenient*" dose, then a child six years old should be able to take seventy-two drops, or three and three-fourths grs., just one-half a fatal dose, in ordinary cases- But *one-half* of a fatal dose of any remedy will be administered, by the discreet physician, *only* in *extraordinary* cases of sickness.

If you will allow me a little more space in your Journal, I will give my views of the use of opium in two or three diseases, and by these views you will see I highly prize opium, not only as a *palliative* but as a *remedy*. But first I will give my rule as a "*safe* and *convenient*" use of laudanum, for infants. It is, for ordinary cases, one drop of laudanum for every *year* in the child's age, to be doubled in a case of severe suffering. This is my direction to "Mothers," but *I* double and quadruple that dose without hesitancy, in a case of *great* emergency, but *never*, I think, did I give a child one year old, *twelve* drops of laudanum, and I hope I never may feel obliged to.

I think the Professor rates the value of morphine too *low*, compared to opium. I offer it merely as my opinion, that one gr. of morphine is fully equal to four of opium.

Now, as to opium in Dysentery. Eleven or twelve years ago last fall, I concluded opium was good in Dysentery, and determined the next case of that disease I saw, I would give it the full benefit of a *full* dose of *ten grs*. But my *first* case was a feeble female, who had been given to the use of Homœopathy for a number of years. For twenty hours previous to my call she had been up as often as every *half* hour, with a *constant* desire to sit on the stool, very severe bearing-down pain, and the passages of blood and mucus. I gave to her *eight* grs. of opium at *once*, and dealt other powders, of opium v grs., quinine iij gr. each, to be taken every three hours through the night, *if she got up as often*. This was a little after dark, and she did not have to leave the bed again till morning, and then to assist about the house, as she had *no* movement of the bowels for a number of days, she required a laxative. Succeeding so well in the treatment of that case as the *first* on my new plan, (I say, *my*, for I never heard of opium being given so freely, in dysentery, before,) I have followed the same plan invariably, of giving eight to ten grs. at *once*, and in most every case have succeeded as well. A very few times, opium has failed me, and *when* it fails, I am completely at a loss *what* to do. I said I *invariably* followed the opium plan of treatment. I do *not* and with *infants* I *dare not*. Hence dysentery in *infants* is the plague of my life.

And to the use of opium in intermittent and bilious fevers, a word:

I think I can safely say, I have not made, for adults, twelve prescriptions in twelve years, for the arrest of an intermittent, remittent, or bilious fever, when quinine was the base of the prescription, but what opium entered *largely* as one of the *remedies.* I *always* use it, and *rely* upon its beneficial effect. In "congestive intermittents," it, with me, stands at the top of the tower, if seen in the stage of congestion. Illustrating my views by the case of Mrs. S., as reported on page 37, I should have preferred for the first prescription, Opii. gr v, Camphor gr. iij, and Ipecac gr. ij, and as soon as re-action had become well developed, quinine and opium at shorter intervals than recommended by Dr. H. though in *that* case the paroxysm, it seems, was promptly arrested by the administration of the remedy at *long* intervals.

I am surprised that Prof. H. did not speak more particularly of the use of opium in the treatment of Pleurities and Pneumonitis, especially of the last mentioned disease. "Walshe on the Heart and Lungs," goes through the treatment of Pneumonitis without so much as intimating that there is such a remedy in the Materia Medica as opium. If a father saw his little family of half a dozen children sinking for the last time, to find a watery grave, and he could save *one*, and *but one*, and *the* one he chose, he would be placed in straightened circumstances in making a selection; so, in selecting from a half dozen of the remedies for the treatment of pneumonitis. But *I* should say, most *emphatically*, let *opium* be *one* of the remedies.

Hoping I may have offered at least *one* beneficial thought,

I remain, very truly and sincerely, yours,

J. Blount, M. D.

—

The above criticism, in one point, at least, is justly liable to *criticism*, for 240 drops of laudanum is equivalent to *two* fluid drachms, not *four*, as stated; the writer evidently based his calculation on minims instead of drops.

